# Increased gut microbiota diversity in women with uterine fibroids: Insights from a pilot study on gut and reproductive tract microbiota

**DOI:** 10.1371/journal.pone.0327177

**Published:** 2025-07-24

**Authors:** Lidia Korczyńska, Michalina Dąbrowska, Maria Kulecka, Piotr Olcha, Tomasz Łoziński, Maciej Brązert, Jerzy Ostrowski, Ewa E. Hennig, Michał Ciebiera, Natalia Zeber-Lubecka

**Affiliations:** 1 2nd Department of Obstetrics and Gynecology, Center of Postgraduate Medical Education, Warsaw, Poland; 2 Department of Genetics, Maria Sklodowska-Curie National Research Institute of Oncology, Warsaw, Poland; 3 Department of Gastroenterology, Hepatology and Clinical Oncology, Centre of Postgraduate Medical Education, Warsaw, Poland; 4 Department of Gynecology and Gynecological Endocrinology, Medical University of Lublin, Lublin, Poland; 5 Department of Gynecology and Obstetrics, Institute of Medical Sciences, Medical College of Rzeszow University, Rzeszow, Poland; 6 Development and Research Center of Non-Invasive Therapies, Pro-Familia Hospital, Rzeszow, Poland; 7 Department of Obstetrics and Gynecology, Pro-Familia Hospital, Rzeszow, Poland; 8 Department of Infertility and Reproductive Endocrinology, Poznan University of Medical Sciences, Poznan, Poland; Yakin Dogu Universitesi, TÜRKIYE

## Abstract

Uterine fibroids (UFs) are still mysterious lesions, they are influenced by hormonal imbalances and chronic inflammation, with recent emerging evidence suggesting a role for microbiota. While gastrointestinal and vaginal microbiota in UF patients have been moderately explored, this study uniquely examines endometrial microbiota in women with UFs. Aim of this study was to investigate the microbiota composition in the uterine cavity, cervix and stool using 16S rRNA bacterial gene sequencing, alongside the concentration of bacterial metabolites in stool samples, comparing women with UFs to a control group. Results revealed no statistically significant differences in α- and β-diversity in cervical swab and endometrial tissue samples between patients with UFs and controls. However, detailed analyses highlighted the overrepresentation of *Lactobacillus iners* in cervical samples of patients with UFs, a species often associated with vaginal dysbiosis. Gut microbiota analysis demonstrated increased Shannon index measured α-diversity in patients with UFs, yet no differences in richness or β-diversity. While short-chain fatty acids (SCFAs) modulate inflammation and immunity, this study found no significant differences in SCFA or amino acid levels, though trends warrant further investigation. The small sample size and microbiota variability limited statistical significance, emphasizing the need for larger studies to unravel microbiota’s complex role in UF pathogenesis. In conclusion, the study underscores microbiota’s potential impact on gynecological health and highlights avenues for future research, including microbiome-targeted therapies for UFs and related disorders.

## Introduction

Uterine fibroids (UFs) are the most common benign tumors in women of reproductive age. They may affect even over 70% of women worldwide [1]. The risk factors for UFs include age, obesity, positive familial history, low levels of vitamin D, and endogenous and exogenous hormonal factors. UFs are heterogeneous as regards their number, composition and size [2]. They may require surgical treatment and are a major source of gynecological and reproductive dysfunction [1]. UFs constitute a significant health impact for the affected women and a financial burden for healthcare systems.

The human gut hosts approximately 100 trillion bacteria that play a key role in digestion and nutrient absorption [3–5]. Numerous intestinal microbes influence the physiological functions of the host and affect the synthesis and secretion of hormones, trace elements, growth factors and immune system functions [6]. The intestinal flora may be modified by hormonal interactions both in vitro and in vivo affecting the biological balance of the body [7]. UFs, which are hormone-dependent tumors, are primarily influenced by estrogen and progesterone [8]. Several studies indicate that the gut microbiota are involved in estrogen metabolism through enterohepatic circulation [9]. Disruptions in the composition of the gut microbiome may affect hormone levels, potentially influencing the development and growth of UFs. Changes in the gut microbiome can alter the reabsorption and excretion of estrogen, impacting estrogen-dependent conditions such as UFs [10]. The results suggested that the systemic distribution of gut bacteria extended to the patients’ fibroids, likely following dysbiosis or impaired intestinal barrier [10].

The use of next-generation sequencing (NGS) of hypervariable fragments of the 16S rRNA bacterial gene revealed the presence of numerous microorganisms within the uterine cavity, which were usually found in the vagina and in the colon [11,12]. However, compared to the microbiomes of the uterine cervix and vagina, the level of the bacterial colonization of the uterine cavity is relatively low [13]. Previous research allowed the determination of the so-called “healthy” microbiome of the vaginal and uterus in women of reproductive age, with the dominant *Lactobacillus* (LD, *Lactobacillus*-dominant) species, with the abundance of *Lactobacillus spp*. above 90% and without the dominance of *Lactobacillus* (NLD, non-*Lactobacillus* dominant), with the abundance of *Lactobacillus spp*. below 90% [14]. Hormonal changes affect the vaginal and endometrial microbiota. Exogenous progestogens significantly alter the microbiota of the endometrium, including the reduction of the diversity of *Lactobacillus spp.* [15].

To date, there is a scarcity of studies investigating the profiles of gut and uterine microbiota in women with UFs. Moreover, to the best of our knowledge, no research has comprehensively analyzed the microbial composition of endometrial tissues in this patient population. Existing literature has largely overlooked the potential relationship between site-specific microbial dysbiosis and the pathophysiology of UFs, particularly in relation to bacterial metabolites. Addressing this gap, our study offers a novel and integrated approach by analyzing both local (uterine cavity and cervix) and systemic (gut) microbiota, alongside microbial metabolites. Therefore, the aim of this study was to comprehensively characterize the microbiota composition at three anatomical sites – the uterine cavity, cervix, and stool – as well as to quantify selected stool bacterial metabolites in women with UFs compared to fibroid-free controls, in order to identify potential microbiota differences associated with the presence of uterine fibroids.

## Materials and methods

### Patient characteristics

The study was conducted in accordance with the guidelines of the Helsinki Declaration of 1964 and approved by the Bioethics Committee at the Center of Postgraduate Medical Education, no. 63/2022. All participants gave their informed consent to participate in the study. The study was conducted in cooperation with the 2nd Department of Obstetrics and Gynecology based in the Warsaw Institute of Women’s Health and the Department of Gastroenterology, Hepatology and Clinical Oncology, Center of Postgraduate Medical Education in Warsaw.

The study group included women of Polish origin (Caucasian race) who had given written informed consent before being included in the study. The inclusion criteria were: women aged over 18 with gynecological indications for hysteroscopy. The exclusion criteria were: pregnancy and lactation, a history of bowel surgery requiring an intestinal stoma, chronic immunosuppression, oncological treatment, contraception, menopausal hormone therapy, hormonal treatment for endometrial hyperplasia up to one month before the procedure, hormonal intrauterine device (IUD), abnormal cervical cytology result, use of antibiotics up to 30 days before the procedure. The patients were recruited between June 2022 and June 2023. The study group included patients with UFs who underwent hysteroscopy. The control group included patients without UFs requiring hysteroscopy for other reasons, such as cervical and uterine polyps, abnormal bleeding from the genital tract or infertility.

In patients undergoing hysteroscopy, specimens of the endometrium covering a possible pathological lesion in the uterine cavity and specimens of the normal endometrium located at a distance of at least 1 cm from any pathology of the uterine cavity were collected into sterile Eppendorf tubes. Prior to hysteroscopy, cervical swabs were collected from patients using 4N6FLOQSwabs™ (Thermo Fisher Scientific, USA). Endometrial samples were collected using sterile, single-use sampling brushes under direct visualization with the aid of a sterile speculum. Prior to insertion, the cervical os was gently cleansed with sterile saline to reduce potential contamination from vaginal or cervical flora. The sampling instrument was carefully inserted transcervically without contact with the vaginal wall. All procedures were performed by experienced gynecologists following strict aseptic protocols. To monitor for contamination, blank swabs (negative controls) were collected during each sampling session and processed alongside clinical samples. Moreover, the patients were asked to provide a stool sample in sterile containers. The collected material was secured and frozen at -20^o^C.

### 16S rRNA bacterial gene sequencing and bioinformatic analysis

Genomic bacterial DNA was isolated from the collected uterine tissues and cervical swabs using the commercial QIAamp DNA Mini Kit (Qiagen, Germany). The QIAamp Fast DNA Stool Mini Kit (Qiagen, Germany) was used to isolate DNA from fecal material [6]. Subsequently, the construction of bacterial 16S rRNA gene libraries was created using the Ion 16S™ Metagenomics Kit (Thermo Fisher Scientific, USA) and the Ion Plus Fragment Library Kit (Thermo Fisher Scientific, USA). As part of contamination control, negative extraction controls and blank swabs were included during DNA extraction and library preparation. Additionally, a commercially available mock microbial community (ZymoBIOMICS Microbial Community Standard, Zymo Research, USA) was used as a positive control to validate sequencing accuracy and detect potential technical biases. Internal control steps were also included during library construction to monitor quality and ensure reproducibility. The constructs were sequenced using Ion Torrent technology with the Personal Genome Machine (Thermo Fisher Scientific, USA) using PGM™ Hi-Q™ View Sequencing Kit reagents (Thermo Fisher Scientific, USA), as described previously [16].

Then, a bioinformatic analysis was performed to assess the composition of the cervical microbiota, endometrial tissue biofilm and stool. Raw BAM files were transformed into FASTQ format using the SamToFastq utility from the Picard toolkit. Subsequent analyses were performed using Mothur version 1.43. The resulting FASTQ files were then converted into the PASTA format for further processing [17]. Sequences ranging from 200 to 300 base pairs in length were selected, ensuring an average base quality score of at least 20 within a 50-base sliding window and limiting homopolymer runs to a maximum length of 10. Chimeric sequences were detected using the vsearch algorithm with default settings, referencing an internal sequence database [18]. Chimeric sequences were excluded, and the remaining 16S rRNA sequences were taxonomically classified employing the Wang method with the SILVA bacterial 16S rRNA database as the reference [19]. The bootstrap threshold was set at 80%. Alpha-diversity analysis was performed using the Shannon and Chao indices [20]. Principal Coordinate Analysis (PCoA) was conducted utilizing the Bray-Curtis distance metric [21]. The significance of the observed clustering patterns was evaluated using the ANOSIM test. The LinDA approach, applied with default configuration, was used to evaluate taxonomic abundance differences [22]. The Mann-Whitney U test was used to assess differences in diversity indices between control samples and fibroid samples, while the paired Wilcoxon test was used to identify statistically significant differences in paired patient samples. After controlling for the false discovery rate (FDR), adjusted *p*-value (*p*_adj_) of less than 0.05 was viewed as significant. Species-level identification of Lactobacillus was achieved by mapping the reads against the Greengenes v13_8 reference database [23].

### Stool metabolome analysis

In addition, concentrations of bacterial metabolites, including short chain fatty acids (SCFAs) and amino acids (AAs) were examined using gas chromatography mass spectrometry (GC-MS). A 100 mg portion of stool was placed into a 2 ml tube containing ceramic beads (Ohaus Corporation, Parsippany, NJ, USA), specifically intended for environmental sample analysis, and then thoroughly homogenized using mechanical disruption. The study used commercial calibration standards for SCFAs (formic acid, acetic acid, propanoic acid, butyric acid, isobutyric acid, pentanoic acid, isocaproic acid, and hexanoic acid) and AAs (alanine, glycine, valine, leucine, isoleucine, proline, methionine, phenylalanine, tyrosine) (Sigma-Aldrich, USA). Derivatization of both samples and standards was performed using isobutyl chloroformate as the reagent [24].

The analysis was conducted using a GC 7890 system coupled with an Agilent 7000D Triple Quadrupole MS and G4513A autosampler, utilizing a VF-5ms capillary column (30 m × 0.25 mm × 0.50 μm). Data acquisition in full scan mode (m/z 15–650) was performed at 4.9 scans/s and processed using MassHunter software (Agilent Technologies, USA) [24].

## Results

### Patient characteristics

We recruited 55 patients for the study. Finally, material collected from 51 patients, including 29 from the group with uterine fibroids (UFs) and 22 from the control group, met the qualitative and quantitative criteria for inclusion in further analysis. During recruitment, a total of 28 and 19 cervical swabs, 21 and 16 samples of pathologically altered endometrial tissue, and 25 and 17 samples of normal endometrial tissue were collected from UF patients and the control groups, respectively, as well as 8 stool samples from each group. The clinical characteristics of patients participating in the study are presented in Table 1. The mean age of participants was 44.9 ± 11.01 years in the UF group and 45.2 ± 11.37 years in the control group. Median age was 44.1 and 44.0 years, respectively. The majority of patients in both groups were premenopausal; specifically, 26 out of 29 UF patients and 20 out of 22 control patients were premenopausal. Three UF patients and two control patients were perimenopausal. None of the participants were postmenopausal. Use of hormonal contraception within the last 12 months was reported by 5 patients in the UF group and 4 in the control group. Polycystic ovary syndrome (PCOS) or endometriosis were noted in 2 patients with UFs; neither diagnosis occurred in the control group. Seven patients in each group had never been pregnant. Primiparity was reported by 13 patients in the UF group and 11 in the control group. At least one miscarriage was reported by 6 UF patients and 5 control patients. Various forms of infertility treatment had been conducted in 6 UF patients and 2 from the control group. A history of inflammation (e.g., recurrent vaginal infections, adnexitis, endometritis) was reported by 7 patients with UFs and 4 in the control group. Treatment with antibiotics up to 6 months prior to the procedure was reported by 6 patients in the UF group and 4 in the control group. The antibiotics listed by the patients included: rifaximin, azithromycin, amoxicillin with clavulanic acid, cefuroxime, metronidazole, and tetracycline. Vitamin D supplementation was noted in 17 UF patients and 9 control patients. A total of 21 patients with UFs and 13 in the control group had a history of procedures or surgeries, including abdominal laparoscopic surgeries, laparotomies due to gynecological or surgical indications, gynecological procedures such as hysteroscopies or curettages, as well as soft tissue operations. To reduce potential confounding, control participants were recruited to closely match UF patients in terms of age and reproductive history, and were confirmed to be free from fibroids or other uterine pathologies via clinical and ultrasonographic examination.

**Table 1:** Characteristics and size of the study and control group.

	N
**Study group (UFs)**	
**Uterine fibroids**	27
**Uterine fibroids + adenomyosis**	1
**Uterine fibroids + infertility**	1
**Total**	29
**Age**	
**Median**	44.1
**Mean**	44.9
**SD**	11.01
**Control group**	N
**Polyps**	18
**Uterine adhesions**	1
**Abnormal bleeding from the genital tract**	1
**Abnormal endometrium on ultrasound**	2
**Total**	22
**Age**	
**Median**	44.0
**Mean**	45.2
**SD**	11.37

UF, uterine fibroid; SD, standard deviation.

### 16S rRNA bacterial gene sequencing

**Fig 1:**
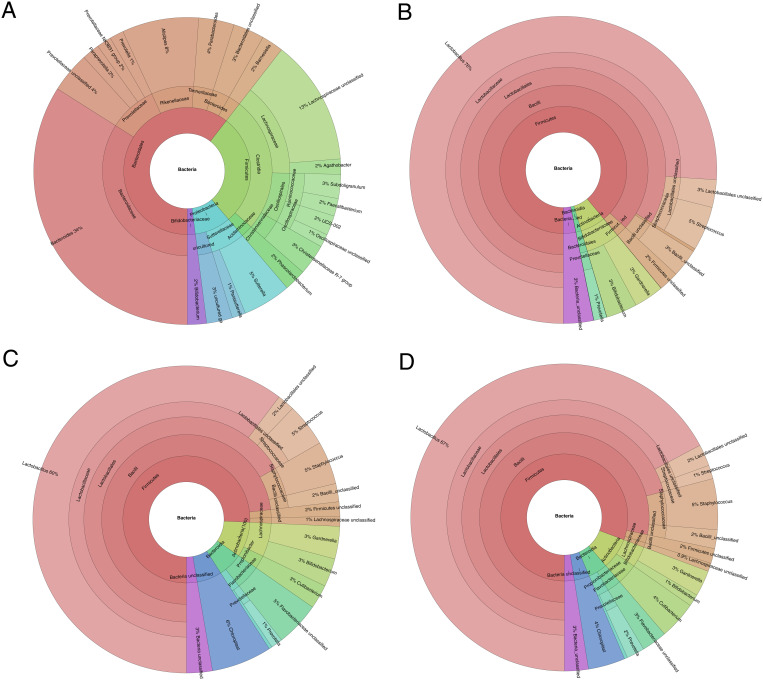
Krona charts of the taxon abundance with a mean >1% of the total reads found in the (A) stool samples, (B) cervical swabs, (C) pathologically altered and (D) lesion-free tissues.

There were, on average, 88954, 90347, 67819 and 45494 sequencing reads generated for cervical swabs, normal endometrium tissues, pathologically altered endometrium tissues and stool samples, respectively. From 2666 (normal endometrium tissue) to 641 (stool) bacterial species were present, with 383 (normal endometrium tissue) to 102 (cervical swab) species being present in more than 0.01% of reads (Fig 1).

**Fig 2:**
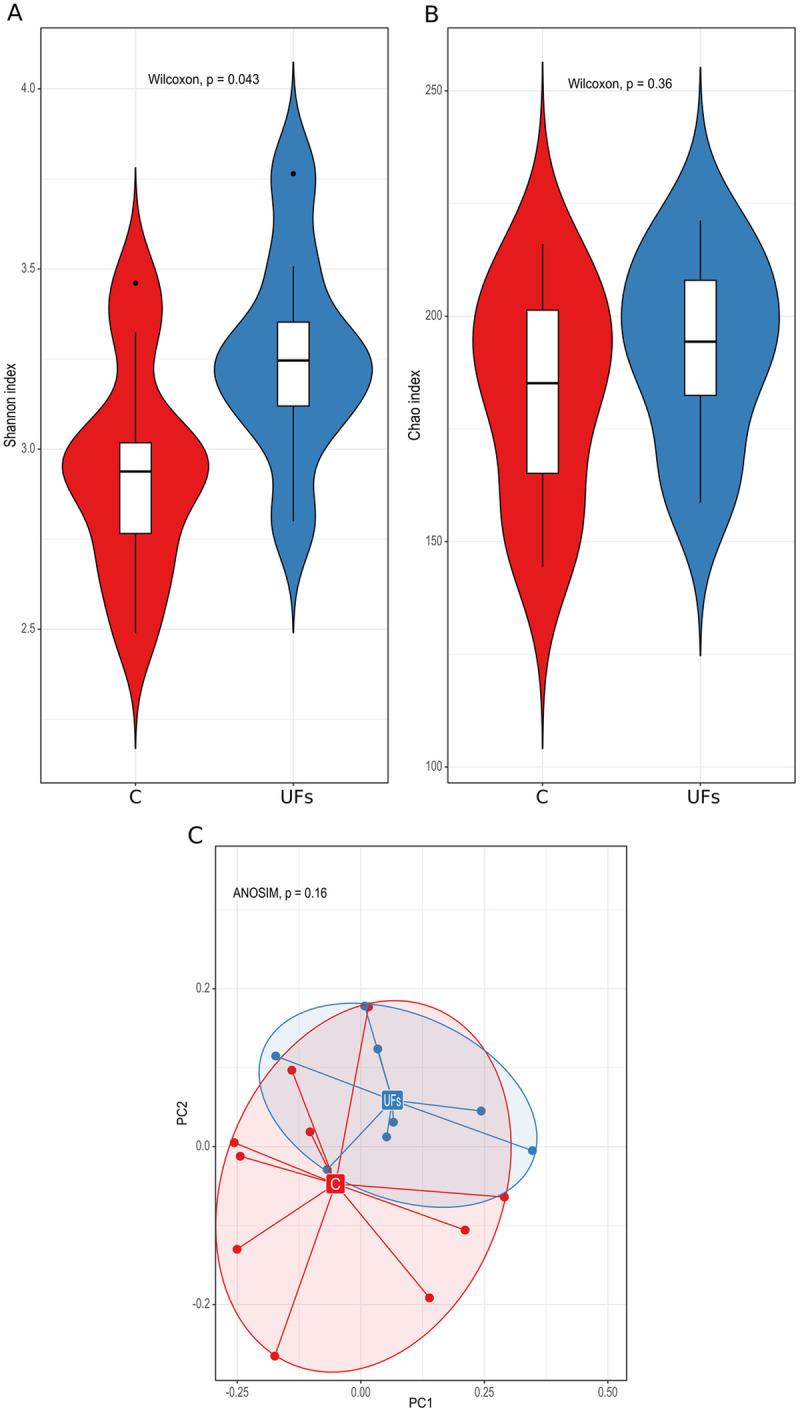
(A) The Shannon and (B) Chao indices and (C) the analysis of the main components based on the Bray–Curtis index of the stool microbiota in the uterine fibroid (UF) and control (C) groups.

The 16S rRNA bacterial gene sequencing of DNA isolated from stool samples showed significantly higher bacterial α-diversity, as measured by the Shannon index (*p* = 0.043), in the group of women with UFs compared to the control group (Fig 2A). However, we found no significant differences between the groups both in terms of the richness of bacterial species measured with the Chao index (Fig 2B) and their β-diversity (Fig 2C). We identified 15 species differentiating the group of women with UFs from the control group at the level of *p*-value < 0.05 significance. Eight and seven of them were more abundant (*Prevotellaceae NK3B31 group*) and less abundant (*Bifidobacterium*), respectively, in women with UFs (S1 Table). However, for none of the identified species statistical significance reached the level of the *p*_adj_-value < 0.05.

**Fig 3:**
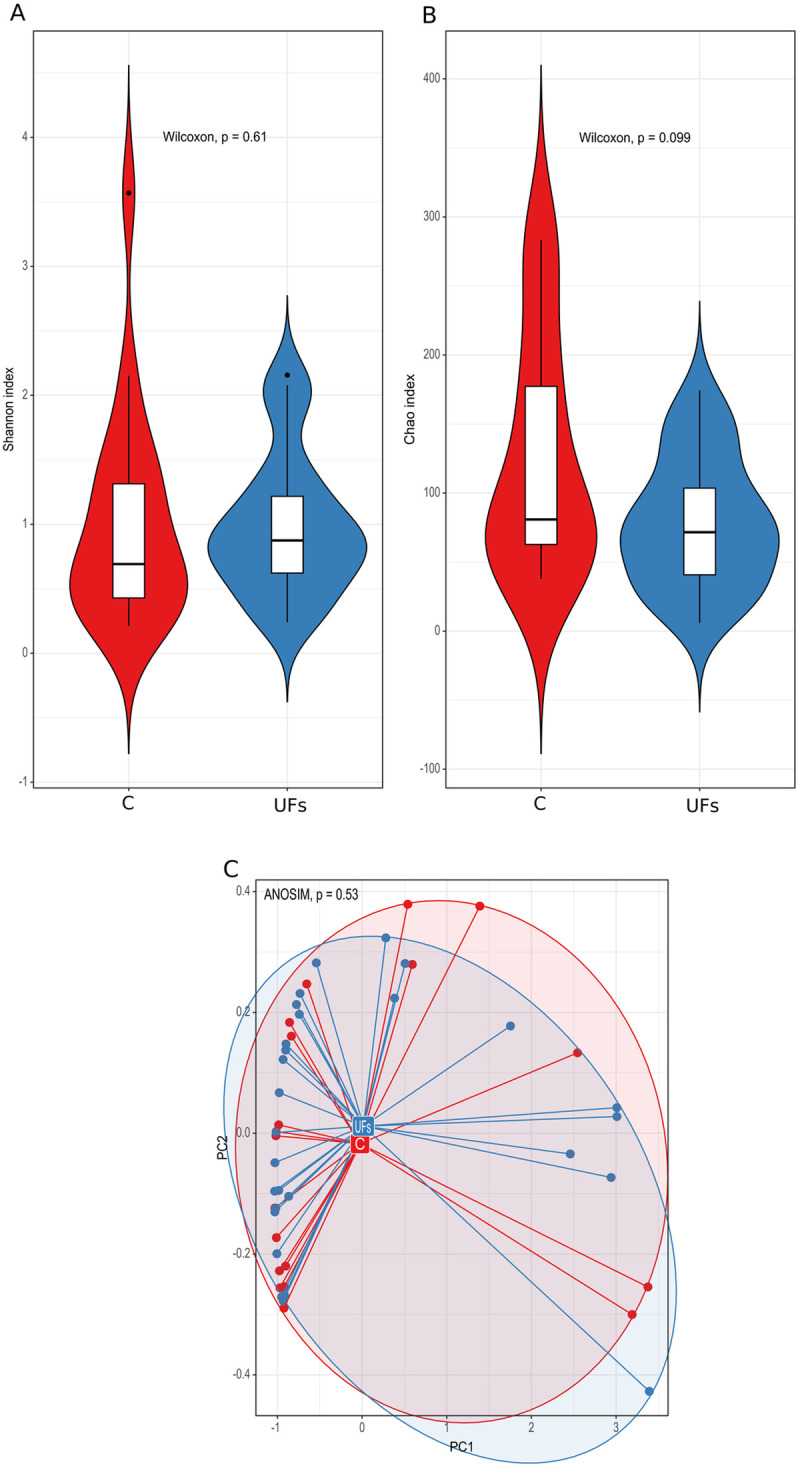
(A) The Shannon and (B) Chao indices and (C) the analysis of the main components based on the Bray–Curtis index of the cervical microbiota in the uterine fibroid (UF) and control (C) groups.

When analyzing samples of cervical swabs, no significant difference between the groups at both α- and β-diversity levels was shown (Fig 3). The identified possibly differentiating bacteria (significance at *p* < 0.05, without adjustment), were less abundant in swab samples obtained from women with UFs than control group (S2 Table). However, searching for the *Lactobacillus* species colonizing the cervical canal, we found that *Lactobacillus iners* was significantly (*p*_adj_ = 0.05; *p *= 0.0035) overrepresented in the cervix of women with UFs.

**Fig 4:**
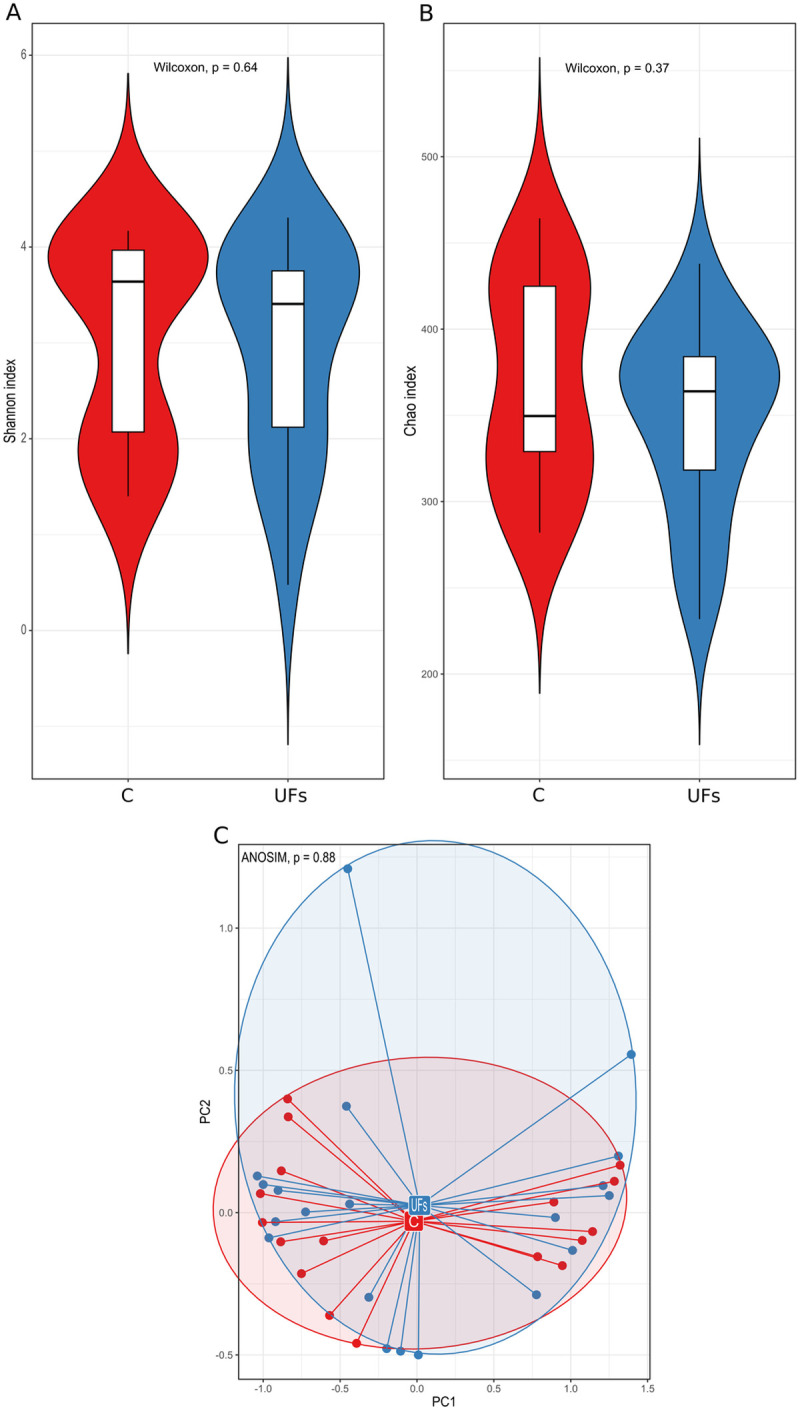
(A) The Shannon and (B) Chao indices and (C) the analysis of the main components based on the Bray–Curtis index of the pathologically altered tissue in the uterine fibroid (UF) and control (C) groups.

Subsequently, we compared the microbiota inhabiting the endometrial tissue. This was the first time that the microbiome composition of pathologically altered endometrial tissues was compared between a group of women with UFs and the control group. Similarly to cervical microbiome, no significant difference between the groups was shown at both α- and β-diversity levels (Fig 4), However, 62 bacterial species were identified differentiating pathologically altered tissue samples of women with UFs and the control group, at the level of *p* < 0.05 significance. Of these, 22 (*Atopobium, Ezakiella*) and 40 (*Ilumatobacter, Rhodocyclaceae C39*) species were more and less represented in the group of women with UFs, respectively (S3 Table). Among *Lactobacillus* species, *Lactobacillus curvatus*, was less abundant in endometrial tissues from patients with UFs, but at the significance level without adjustment.

**Fig 5:**
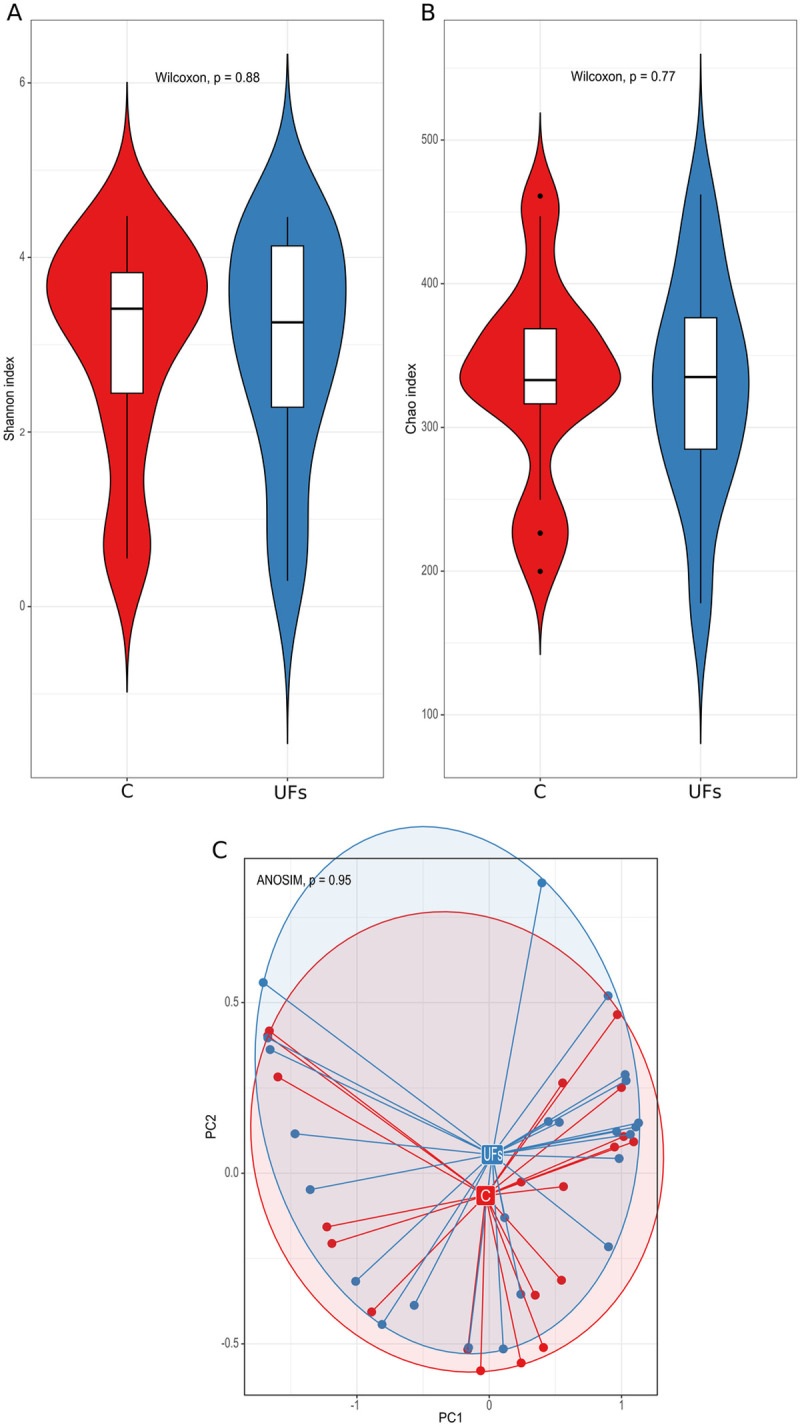
(A) The Shannon and (B) Chao indices and (C) the analysis of the main components based on the Bray–Curtis index of the lesion-free tissue altered tissue in the uterine fibroid (UF) and control (C) groups.

Similarly to the comparison of the pathologically altered endometrial tissue, no significant intergroup differences were noted as regards the lesion-free endometrial tissue both at the level of the α- and β-diversity (Fig 5). Also, at the level of *p* < 0.05 statistical significance, we identified 28 species which differentiated the lesion-free tissue samples from women with UFs and control group. Half of them were more abundant (*Atopobiaceae unclassified*) and half were less abundant (*Acidovorax*) in the group of women with UFs (S4 Table). In this tissue, we found no *Lactobacillus* species significantly differentiating the UF patients and control groups.

### Stool metabolome analysis

**Fig 6:**
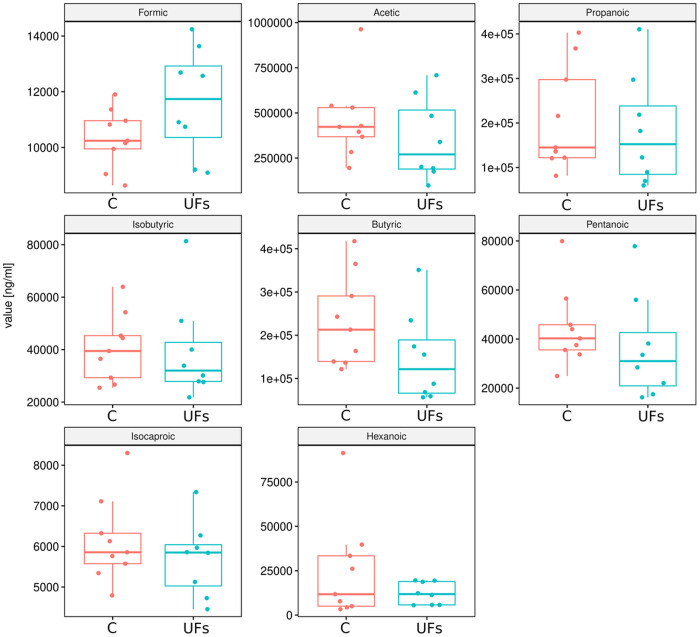
Changes in SCFA concentrations in stool samples obtained from patients with uterine fibroids compared to the control group.

**Fig 7:**
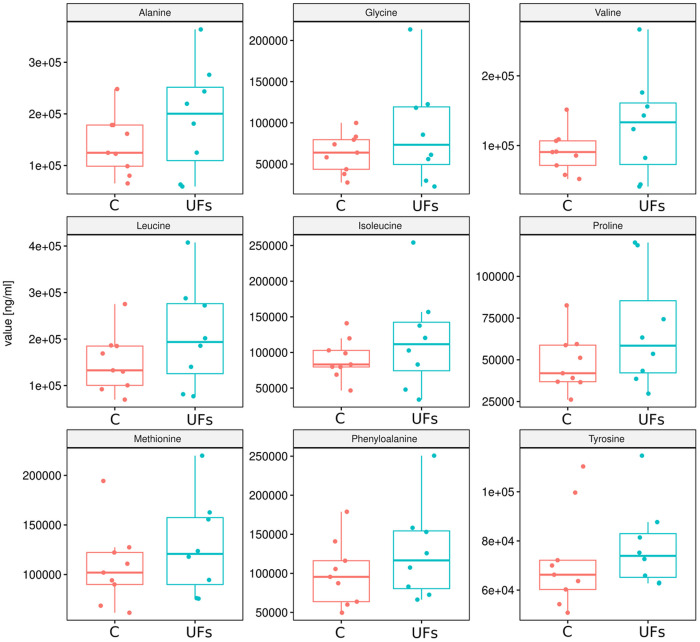
Changes in AA concentrations in stool samples obtained from patients with uterine fibroids compared to the control group.

Next, we compared the relative concentrations of stool metabolites between UF patient and control groups. However, no statistically significant differences were obtain between the compared groups, both for SCFAs (Fig 6) and AAs (Fig 7). Nevertheless, a distinct trend of either increasing or decreasing concentrations of the studied metabolites was observed.

## Discussion

The microbiota of the cervix, vagina, and gut is increasingly recognized as a potentially critical factor in gynecological health, with microbial imbalances, or dysbiosis, playing a role in various conditions [25,26]. While extensive research was conducted on the microbiota of the gastrointestinal tract, oral cavity, and the skin, the study of microbial communities [27,28] within the female reproductive system is still at its early stages and there are still numerous unknowns [29]. In conditions like endometriosis, it was revealed that dysbiosis might contribute to inflammation, immune response disruptions, and disease progression [30]. In contrast, studies on the microbiota in UFs are scarce, and the available research presented inconsistent findings on the microbial composition [10,12,31–33]. Therefore, the aim of this study was to assess the microbiota composition at three locations: the uterus cavity, cervix, and stool, as well as to measure the concentration of bacterial metabolites in the stool of women with UFs compared to UF-free women in the control group.

There are relatively few studies focusing on the gut and vaginal microbiota of UF patients [31–34]. However, to date, no studies have analyzed the composition of the endometrial microbiota in this disease. Although, the results of our study did not show statistically significant differences in bacterial α- and β-diversity in samples of cervical swabs and endometrial tissues obtained from women with UFs compared to the control group, some findings still deserve discussion. Zhang et al. [31] showed increased vaginal microbiota diversity in patients with UFs in a pre-operative high-intensity focused ultrasound (HIFU) group. Moreover, HIFU treatment significantly decreased the abundance and diversity of the vaginal microbiome in UF patients [31]. It should be noted that the inconsistency in our results and that study may arise from several factors, such as the use of different methods for assessing microbiota composition, the fact that previous analyses were conducted only in Asian populations, and variations in group sizes, which also affect the ability to obtain statistically significant results.

Also, we acknowledge that none of the bacteria identified in the comparison between group of women with UFs and UF-free controls, differentiated either cervical swab or endometrial tissue samples with the *p*_adj_ significance of less than 0.05, commonly accepted as required in microbiome studies. However, considering the limited number of samples analyzed and the pilot nature of the study, the indication of statistically significant differences at *p* < 0.05 without correction may be worth noting as potentially differentiating bacteria. Interestingly, upon a detailed identification of *Lactobacillus* species, *Lactobacillus iners* was found to be overrepresented in swabs from women with UFs compared to the control group.

In contrast to other *Lactobacillus* species, such as *Lactobacillus crispatus*, *L. iners* is not always considered beneficial for health. It has a less robust ability to prevent pathogenic overgrowth compared to other species. *L. iners* produces a smaller amount of lactic acid and has a thinner cell wall, making it potentially less effective as a barrier against pathogens [35,36]. These bacteria can survive under altered vaginal environmental conditions, such as higher pH levels, which may facilitate the development of pathogens. This distinguishes it from other *Lactobacillus* species that prefer a more stable, acidic environment [37]. The presence of *Lactobacillus iners* is often correlated with vaginal dysbiosis, such as bacterial vaginosis (BV). Research showed that in patients with BV, *L. iners* was one of the dominant species, suggesting that its presence might not offer protection against the development of BV [37]. Some authors suggested that *L. iners* might facilitate the transition between a healthy microbiota and a state of dysbiosis [35]. Given its ability to persist in unstable environments, modulate local immunity, and potentially compromise epithelial integrity, one could hypothesize that *L. iners* contributes to a microenvironment conducive to chronic low-grade inflammation [38,39]. This, in turn, may influence hormonal signaling or local immune responses in the uterine environment, potentially favoring the development or progression of UFs [32]. While further mechanistic studies are needed, our findings suggest that *L. iners* may be more than a passive marker of dysbiosis – it could play an active role in shaping conditions relevant to uterine health. This indicates that *L. iners* could serve as a marker of vaginal microbiota instability, and its presence at higher quantities may be a warning sign for potential disruptions, even if it is not the direct cause of the disorders [40]. Moreover, Robbins et al. [32] showed that vaginal bacterial profiles dominated by *L. iners* and with low *Lactobacillus* content were more likely to be associated with UFs compared to other *Lactobacillus*-dominated profiles, although those results were not statistically significant. In our opinion, the diversities connected with *Lactobacilli* might be of interest for future studies.

The results of studies conducted so far have indicated a connection between changes in gut microbiome diversity and various gynecological diseases, including endometriosis. However, the results are inconsistent [30]. In our study, higher Shannon index measured α-diversity of bacteria in stool samples obtained from patients with UFs was showed compared to the control group, with no observed differences either in the richness or β-diversity. In contrast, the results by Mao et al. [33] revealed that α-diversity was significantly lower in patients with UFs than that in healthy controls and the gut microbial composition in UF patients differed from that in the healthy control group. Given the known high interindividual variability of the gut microbiota, identifying consistent and disease-specific microbial patterns requires a sufficiently large and well-matched cohort. In smaller pilot studies such as ours, this variability can mask subtle but biologically meaningful associations. Therefore, discrepancies between our findings and those of other researchers, such as Mao et al., may be partially attributed to differences in cohort size, demographic composition, and methodological approaches [33].

The gut microbiota influences estrogen levels by producing β-glucuronidase, an enzyme that converts estrogens into their active forms through deconjugation [9]. Estrogens are hormones of key significance in the development of UFs, although progesterone is considered more important [41,42]. Excessive estrogen or estrogen-progesterone imbalances appear to be contributing factors [12]. With impaired gut microbiome, the process of detoxification and excretion of estrogens may be weakened. The resulting excessive accumulation of estrogens changes the hormonal environment into one that is favorable for UFs occurrence and growth [33,43]. Estrogens influence the diversity of the gut microbiome, thereby reducing the availability of β-glucuronidase and increasing estrogen excretion. Conversely, low estrogen levels were found to reduce the diversity of the gut microbiome, which increased β-glucuronidase activity and estrogen re-circulation [44].

According to some authors, the intestinal microbiome also affects the vaginal microbiome through the so-called gut-vagina axis. Intestinal dysbiosis may lead to disturbances in the vaginal microbiome, which increases the risk of infection and other gynecological problems that may indirectly affect the health of the uterus [45,40]. Changes in the gut microbiome may also influence inflammation and the immune response, which are important factors in the pathogenesis of UFs [46]. A decrease in the abundance of bacteria of the genus Bacteroidetes and an increase in the abundance of Firmicutes and Proteobacteria were noted in stool samples of patients with UFs, which was a typical sign of dysbiosis [25]. Therefore, understanding the role of the gut microbiome and its diversity may be crucial to developing new strategies for the treatment and prevention of UFs, as well as other gynecological disorders, such as through individualized probiotics.

The gut microbiota influences host physiology partly through the production of SCFAs, metabolites that not only remain within the gut but can also cross the gut barrier into the systemic circulation [47,48]. Chadchan et al. demonstrated a reduction in SCFA levels in the feces of mice with endometriosis, and treatment with *n*-butyrate was observed to decrease the growth of endometriotic lesions in those mice [49]. Additionally, a case-control study including 137 women identified a correlation between the serum levels of the metabolite, i.e., 2-hydroxybutyric acid, and an increased risk of endometriosis [50]. Those findings suggested that SCFAs might play a significant role in the onset and progression of endometriosis. In our study, possibly due to the low number of samples analyzed, we did not obtain statistically significant results between the compared groups for either SCFAs or AAs, although a certain trend of an increase or decrease in the concentrations of the studied metabolites was noticeable. We acknowledge that the lack of statistical significance in these comparisons weakens the strength of evidence for a consistent association between microbiota-derived metabolites and UFs. However, given the exploratory scope of this pilot study, our intention was to identify potential trends that could inform and refine the design of larger follow-up investigations.

It is also important to note that this study relied on 16S rRNA gene sequencing, which, while useful for taxonomic profiling of microbial communities, provides limited insights into the functional roles or metabolic activities of the identified bacteria. This method does not capture transcriptomic or metabolomic activity, which may be essential for understanding the mechanisms by which the microbiota might influence the development or progression of UFs. Future studies incorporating metagenomic, metatranscriptomic, and metabolomic approaches are warranted to gain a more comprehensive view of microbiota function and its potential interaction with host physiology.

Most of the results obtained in this study were statistically insignificant, which may be attributed to several important limitations. Firstly, the sample size was relatively small, which inherently limits the statistical power of the analyses and increases the risk of Type II errors – that is, the possibility of failing to detect genuine effects or associations due to insufficient sensitivity. As this was a pilot study, our primary goal was to explore potential differences and generate hypotheses for future research, rather than to draw definitive conclusions. Secondly, the microbiota in different anatomical niches – such as the uterine cavity, cervix, and colon – exhibits substantial interindividual variability in terms of bacterial composition. This variability is influenced by numerous intrinsic and extrinsic factors, including genetics, hormonal status, diet, lifestyle, and environmental exposures. Such diversity, although biologically relevant, complicates statistical interpretation and can obscure subtle patterns, especially in small cohorts. Furthermore, these body sites naturally harbor complex and diverse microbial communities. The inherent richness and heterogeneity of these populations may lead to wide dispersion in the data, making it difficult to identify consistent microbial signatures associated with UFs. These limitations underscore the exploratory nature of the present findings and highlight the importance of validating observed trends – such as increased gut microbiota diversity in women with UFs - in larger, well-powered studies. As this was a cross-sectional pilot study, dynamic changes in the microbiota over time or in response to treatment were not assessed. However, longitudinal studies will be necessary to determine how microbial profiles evolve with disease progression or therapeutic interventions.

## Conclusion

Despite the predominantly negative findings, our study identified several noteworthy outcomes that underscore the potential of this research path. Specifically, *Lactobacillus iners* was fund to be overrepresented in cervical swab samples from women with UFs, and stool samples from patients with UFs exhibited higher Shannon-indexed α-diversity compared to the control group. These observations suggest a possible link between microbial diversity and UFs, offering a valuable starting point for further exploration. While these findings are preliminary, they highlight the importance of continuing research in this area. Investigating the role of the microbiome in UFs may provide deeper insights into their pathogenesis, open new diagnostic opportunities, and potentially lead to innovative therapeutic strategies. This promising direction deserves sustained attention and more comprehensive studies. Future research should include larger, demographically homogeneous cohorts and apply longitudinal study designs to monitor microbiota dynamics over time and in response to treatment. Incorporating multi-omics approaches such as metagenomics, metatranscriptomics, and metabolomics could help elucidate functional microbial roles and host–microbiome interactions. Additionally, studies should account for hormonal status, dietary habits, and other lifestyle factors known to influence microbiota composition. The potential of microbial markers like *L. iners* as indicators of reproductive tract instability or early UF risk also warrants further investigation.

## Supporting information

S1 Table Bacteria differentiating stool samples of patients with fibroids from the control group. (XLSX)

S2 Table Bacteria differentiating cervical samples of patients with fibroids from the control group. (XLSX)

S3 Table Selected bacterial species differentiating pathologically altered endometrial tissues in patients with uterine fibroids compared to the control group, at the level of p without adjustment (*p*_adj_). (XLSX)

S4 Table Bacteria differentiating the lesion free endometrial tissue in patients with uterine fibroids from the control group. (XLSX)

## References

[1] Navarro A, Bariani MV, Yang Q, Al-Hendy A. Understanding the impact of uterine fibroids on human endometrium function. Front Cell Dev Biol. 2021;9:633180. doi: 10.3389/fcell.2021.633180 34113609 PMC8186666

[2] Ciebiera M, Ali M, Prince L, Zgliczyński S, Jakiel G, Al-Hendy A. The significance of measuring Vitamin D serum levels in women with uterine fibroids. Reprod Sci. 2021;28(8):2098–109. doi: 10.1007/s43032-020-00363-8 33108619 PMC8262605

[3] Afzaal M, Saeed F, Shah YA, Hussain M, Rabail R, Socol CT, et al. Human gut microbiota in health and disease: unveiling the relationship. Front Microbiol. 2022;13:999001. doi: 10.3389/fmicb.2022.999001 36225386 PMC9549250

[4] Mafe AN, Iruoghene Edo G, Akpoghelie PO, Gaaz TS, Yousif E, Zainulabdeen K, et al. Probiotics and food bioactives: unraveling their impact on gut microbiome, inflammation, and metabolic health. Probiotics Antimicrob Proteins. 2025:10.1007/s12602-025-10452–2. doi: 10.1007/s12602-025-10452-2 39808399

[5] Jikah AN, Edo GI, Ali ABM, Akpoghelie PO, Yousif E, Isoje EF. The regulatory effects of vitamins on immunity and skin health. Archives Dermatological Res. 2025;317(1):576. 10.1007/s00403-025-04086-1 40095097

[6] Zeber-Lubecka N, Kulecka M, Jagiełło-Gruszfeld A, Dąbrowska M, Kluska A, Piątkowska M, et al. Breast cancer but not the menopausal status is associated with small changes of the gut microbiota. Front Oncol. 2024;14:1279132. doi: 10.3389/fonc.2024.1279132 38327745 PMC10848918

[7] Zhang Y, Chen R, Zhang D, Qi S, Liu Y. Metabolite interactions between host and microbiota during health and disease: which feeds the other? Biomed Pharmacother. 2023;160:114295. 36709600 10.1016/j.biopha.2023.114295

[8] Yang Q, Ciebiera M, Bariani MV, Ali M, Elkafas H, Boyer TG. Comprehensive review of uterine fibroids: developmental origin, pathogenesis, and treatment. Endocrine Rev. 2021. doi: bnab039 10.1210/endrev/bnab039 PMC9277653 34741454

[9] Hu S, Ding Q, Zhang W, Kang M, Ma J, Zhao L. Gut microbial beta-glucuronidase: a vital regulator in female estrogen metabolism. Gut Microbes. 2023;15(1):2236749. doi: 10.1080/19490976.2023.2236749 37559394 PMC10416750

[10] K V, Bhat RG, Rao BK, R AP. The gut microbiota: a novel player in the pathogenesis of uterine fibroids. Reprod Sci. 2023;30(12). 10.1007/s43032-023-01289-7 PMC10691976 37418220

[11] Sirichoat A, Sankuntaw N, Engchanil C, Buppasiri P, Faksri K, Namwat W, et al. Comparison of different hypervariable regions of 16S rRNA for taxonomic profiling of vaginal microbiota using next-generation sequencing. Arch Microbiol. 2021;203(3). 10.1007/s00203-020-02114-4 33221964

[12] Korczynska L, Zeber-Lubecka N, Zgliczynska M, Zarychta E, Zareba K, Wojtyla C. The role of microbiota in the pathophysiology of uterine fibroids – a systematic review. Front Cell Infect Microbiol. 2023;13:1177366. 37305407 10.3389/fcimb.2023.1177366 PMC10250666

[13] Ichiyama T, Kuroda K, Nagai Y, Urushiyama D, Ohno M, Yamaguchi T. Analysis of vaginal and endometrial microbiota communities in infertile women with a history of repeated implantation failure. Reprod Med Biol. 2021;20(3):334–44. 34262402 10.1002/rmb2.12389 PMC8254176

[14] Mitchell CM, Haick A, Nkwopara E, Garcia R, Rendi M, Agnew K, et al. Colonization of the upper genital tract by vaginal bacterial species in nonpregnant women. Am J Obstet Gynecol. 2015;212(5):611.e1–9. doi: 10.1016/j.ajog.2014.11.043 25524398 PMC4754962

[15] Toson B, Simon C, Moreno I. The endometrial microbiome and its impact on human conception. Int J Mol Sci. 2022;23(1):485. 35008911 10.3390/ijms23010485 PMC8745284

[16] Zeber-Lubecka N, Kulecka M, Dabrowska M, Baginska-Drabiuk K, Glowienka-Stodolak M, Nowakowski A, et al. Cervical microbiota dysbiosis associated with high-risk Human Papillomavirus infection. PLoS One. 2024;19(4):e0302270. doi: 10.1371/journal.pone.0302270 38669258 PMC11051640

[17] Schloss PD, Westcott SL, Ryabin T, Hall JR, Hartmann M, Hollister EB, et al. Introducing mothur: open-source, platform-independent, community-supported software for describing and comparing microbial communities. Appl Environ Microbiol. 2009;75(23):7537–41. doi: 10.1128/AEM.01541-09 19801464 PMC2786419

[18] Rognes T, Flouri T, Nichols B, Quince C, Mahé F. VSEARCH: a versatile open source tool for metagenomics. PeerJ. 2016;4:e2584. doi: 10.7717/peerj.2584 27781170 PMC5075697

[19] Silva CJ, Caparica AA, Plascak JA. Wang-Landau monte carlo simulation of the Blume-Capel model. Phys Rev E Stat Nonlin Soft Matter Phys. 2006;73(3 Pt 2):036702. doi: 10.1103/PhysRevE.73.036702 16605693

[20] Kers JG, Saccenti E. The power of microbiome studies: some considerations on which alpha and beta metrics to use and how to report results. Front Microbiol. 2022;12:796025. doi: 10.3389/fmicb.2022.796025 35310396 PMC8928147

[21] Wang Y, Sun F, Lin W, Zhang S. AC-PCoA: adjustment for confounding factors using principal coordinate analysis. PLoS Comput Biol. 2022;18(7):e1010184. doi: 10.1371/journal.pcbi.1010184 35830390 PMC9278763

[22] Zhou H, He K, Chen J, Zhang X. LinDA: linear models for differential abundance analysis of microbiome compositional data. Genome Biol. 2022;23(1):95. doi: 10.1186/s13059-022-02655-5 35421994 PMC9012043

[23] DeSantis TZ, Hugenholtz P, Larsen N, Rojas M, Brodie EL, Keller K, et al. Greengenes, a chimera-checked 16S rRNA gene database and workbench compatible with ARB. Appl Environ Microbiol. 2006;72(7):5069–72. doi: 10.1128/AEM.03006-05 16820507 PMC1489311

[24] Kulecka M, Zeber-Lubecka N, Bałabas A, Czarnowski P, Bagińska K, Głowienka M, et al. Diarrheal-associated gut dysbiosis in cancer and inflammatory bowel disease patients is exacerbated by Clostridioides difficile infection. Front Cell Infect Microbiol. 2023;13:1190910. doi: 10.3389/fcimb.2023.1190910 37577378 PMC10413277

[25] Elkafas H, Walls M, Al-Hendy A, Ismail N. Gut and genital tract microbiomes: dysbiosis and link to gynecological disorders. Front Cell Infect Microbiol. 2022. https://www.frontiersin.org/journals/cellular-and-infection-microbiology/articles/10.3389/fcimb.2022.1059825/full 10.3389/fcimb.2022.1059825 PMC9800796 36590579

[26] Okolie MC, Edo GI, Ainyanbhor IE, Jikah AN, Akpoghelie PO, Yousif E, et al. Gut microbiota and immunity in health and diseases: a review. ProcIndian Natl Sci Acad. 2024;91(2):397–414. doi: 10.1007/s43538-024-00355-1

[27] Dieterich W, Schink M, Zopf Y. Microbiota in the gastrointestinal tract. Med Sci. 2018;6(4):116. 10.3390/medsci6040116 PMC6313343 30558253

[28] Li W, Xu X, Wen H, Wang Z, Ding C, Liu X, et al. Inverse association between the skin and oral microbiota in atopic dermatitis. J Invest Dermatol. 2019;139(8):1779-1787.e12. doi: 10.1016/j.jid.2019.02.009 30802424

[29] Kumar L, Dwivedi M, Jain N, Shete P, Solanki S, Gupta R. The female reproductive tract microbiota: friends and foe. Life. 2023;13(6):1313. 37374096 10.3390/life13061313 PMC10301250

[30] Guo C, Zhang C. Role of the gut microbiota in the pathogenesis of endometriosis: a review. Front Microbiol. 2024;15:1363455. doi: 10.3389/fmicb.2024.1363455 38505548 PMC10948423

[31] Zhang P-P, He X-P, Tang W, Chen H-W, Han Y-Y. Alterations in vaginal microbiota in uterine fibroids patients with ultrasound-guided high-intensity focused ultrasound ablation. Front Microbiol. 2023;14:1138962. doi: 10.3389/fmicb.2023.1138962 37138604 PMC10150040

[32] Robbins SJ, Brown SE, Stennett CA, Tuddenham S, Johnston ED, Wnorowski AM, et al. Uterine fibroids and longitudinal profiles of the vaginal microbiota in a cohort presenting for transvaginal ultrasound. PLoS One. 2024;19(2):e0296346. doi: 10.1371/journal.pone.0296346 38315688 PMC10843103

[33] Mao X, Peng X, Pan Q, Zhao X, Yu Z, Xu D. Uterine fibroid patients reveal alterations in the gut microbiome. Front Cell Infect Microbiol. 2022;12. https://www.frontiersin.org/journals/cellular-and-infection-microbiology/articles/10.3389/fcimb.2022.863594/full 10.3389/fcimb.2022.863594 PMC9131877 35646718

[34] Wang W, Li Y, Wu Q, Pan X, He X, Ma X. High-throughput sequencing study of the effect of transabdominal hysterectomy on intestinal flora in patients with uterine fibroids. BMC Microbiol. 2020;20(1):98. doi: 10.1186/s12866-020-01779-7 32299359 PMC7161020

[35] Zheng N, Guo R, Wang J, Zhou W, Ling Z. Contribution of Lactobacillus iners to vaginal health and diseases: a systematic review. Front Cell Infect Microbiol. 2021;11:792787. doi: 10.3389/fcimb.2021.792787 34881196 PMC8645935

[36] Holm JB, Carter KA, Ravel J, Brotman RM. Lactobacillus iners and genital health: molecular clues to an enigmatic vaginal species. Curr Infect Dis Rep. 2023;25(4):67–75. doi: 10.1007/s11908-023-00798-5 37234911 PMC10209668

[37] Zheng N, Guo R, Wang J, Zhou W, Ling Z. Contribution of Lactobacillus iners to vaginal health and diseases: a systematic review. Front Cell Infect Microbiol. 2021;11:792787. doi: 10.3389/fcimb.2021.792787 34881196 PMC8645935

[38] Dabee S, Passmore JAS, Heffron R, Jaspan HB. The complex link between the female genital microbiota, genital infections, and inflammation. Infect Immun. 2021;89(5):e00487-20. doi: 10.1128/IAI.00487-20 PMC8091093 33558324

[39] Glick VJ, Webber CA, Simmons LE, Martin MC, Ahmad M, Kim CH. Vaginal lactobacilli produce anti-inflammatory β-carboline compounds. Cell Host Microbe. 2024;32(11):1897-1909.e7. doi: 10.1016/j.chom.2024.09.001 PMC11694765 39423813

[40] Lehtoranta L, Ala-Jaakkola R, Laitila A, Maukonen J. Healthy vaginal microbiota and influence of probiotics across the female life span. Front Microbiol. 2022;13. 10.3389/fmicb.2022.819958 PMC9024219 35464937

[41] Bulun SE. Uterine fibroids. N Engl J Med. 2013;369(14):1344–55. 24088094 10.1056/NEJMra1209993

[42] Borahay MA, Asoglu MR, Mas A, Adam S, Kilic GS, Al-Hendy A. Estrogen receptors and signaling in fibroids: role in pathobiology and therapeutic implications. Reprod Sci. 2017;24(9):1235–44. doi: 10.1177/1933719116678686 27872195 PMC6344829

[43] Qi X, Yun C, Pang Y, Qiao J. The impact of the gut microbiota on the reproductive and metabolic endocrine system. Gut Microbes. 2022;13(1):1894070. 10.1080/19490976.2021.1894070 PMC7971312 33722164

[44] Lephart ED, Naftolin F. Estrogen action and gut microbiome metabolism in dermal health. Dermatol Ther (Heidelb). 2022;12(7):1535–50. doi: 10.1007/s13555-022-00759-1 35752663 PMC9276867

[45] Gao Y, Shang Q, Wei J, Chen T. The correlation between vaginal microecological dysbiosis-related diseases and preterm birth: a review. Med Microecol. 2021;8:100043.

[46] Wiertsema SP, van Bergenhenegouwen J, Garssen J, Knippels LMJ. The Interplay between the gut microbiome and the immune system in the context of infectious diseases throughout life and the role of nutrition in optimizing treatment strategies. Nutrients. 2021;13(3):886. doi: 10.3390/nu13030886 33803407 PMC8001875

[47] Portincasa P, Bonfrate L, Vacca M, De Angelis M, Farella I, Lanza E, et al. Gut microbiota and short chain fatty acids: implications in glucose homeostasis. Int J Mol Sci. 2022;23(3):1105. doi: 10.3390/ijms23031105 35163038 PMC8835596

[48] Edo GI, Mafe AN, Ali ABM, Akpoghelie PO, Yousif E, Apameio JI, et al. Chitosan and its derivatives: a novel approach to gut microbiota modulation and immune system enhancement. Int J Biol Macromol. 2025;289:138633. 39675606 10.1016/j.ijbiomac.2024.138633

[49] Chadchan SB, Popli P, Ambati CR, Tycksen E, Han SJ, Bulun SE, et al. Gut microbiota-derived short-chain fatty acids protect against the progression of endometriosis. Life Sci Alliance. 2021;4(12):e202101224. doi: 10.26508/lsa.202101224 34593556 PMC8500332

[50] Lefebvre T, Campas M, Matta K, Ouzia S, Guitton Y, Duval G. A comprehensive multiplatform metabolomic analysis reveals alterations of 2-hydroxybutyric acid among women with deep endometriosis related to the pesticide trans-nonachlor. Sci Total Environ. 2024;918:170678. 38316313 10.1016/j.scitotenv.2024.170678

